# Case report: JC polyomavirus nephropathy in simultaneous heart–kidney transplantation: the role of viral-specific *in situ* hybridization staining

**DOI:** 10.3389/fmed.2023.1282827

**Published:** 2023-10-20

**Authors:** Bassam G. Abu Jawdeh, Maxwell L. Smith, Madeline R. Hudson, Girish K. Mour, Pooja Budhiraja, Julie L. Rosenthal

**Affiliations:** ^1^Division of Nephrology, Mayo Clinic Arizona, Phoenix, AZ, United States; ^2^Division of Anatomic Pathology, Mayo Clinic Arizona, Phoenix, AZ, United States; ^3^Department of Pharmacy, Mayo Clinic Arizona, Phoenix, AZ, United States; ^4^Division of Cardiovascular Diseases, Mayo Clinic Arizona, Phoenix, AZ, United States

**Keywords:** polyomavirus, JC virus, immunosuppression, IVIg, *in situ* hybridization

## Abstract

**Introduction:**

JC polyomavirus (JCPyV) is a ubiquitous virus that can be latent in the brain and the kidney. It is the etiologic agent responsible for progressive multifocal leukoencephalopathy, a fatal, demyelinating disease of the central nervous system, and rarely causes polyomavirus nephropathy in immunocompromised kidney transplant recipients.

**Case description:**

We present the first case of JCPyV nephropathy in a simultaneous heart–kidney transplant patient, where viral-specific *in situ* hybridization staining of the kidney tissue was utilized to confirm the diagnosis. The patient was diagnosed 6 years after simultaneous heart–kidney transplantation and was treated with immunosuppression reduction and intravenous immunoglobulin.

**Discussion:**

JCPyV nephropathy should be considered in the differential diagnosis of kidney allograft injury, particularly, with suggestive light microscopy histologic features in the absence of BK polyomavirus viremia and/or viruria. In addition to obtaining JCPyV PCR in the blood, *in situ* hybridization staining may have a utility in confirming the diagnosis. To date, we lack effective JCPyV-specific therapies, and prompt initiation of immunosuppression reduction remains the mainstay of treatment.

## Introduction

Polyomavirus nephropathy occurs in up to 10% of kidney transplant recipients (KTRs) within the first year after transplantation and is often secondary to BK polyomavirus (BKPyV) (1, 2). BKPyV viremia and viruria occur in 20 and 50% of KTR, respectively, and are usually asymptomatic (3). Polyomavirus nephropathy is associated with worse graft outcomes, and to date remains elusive to any effective therapy. Immunosuppression is a major contributor to its pathogenesis. This explains the higher incidence in recent years with the widespread use of the more potent mycophenolate/tacrolimus regimen as the backbone of modern maintenance immunosuppression (3). JC polyomavirus (JCPyV) is another ubiquitous virus that can be latent in the brain and the kidneys (4). It is the etiologic agent responsible for progressive multifocal leukoencephalopathy (PML), a fatal, demyelinating disease of the central nervous system that occurs in immunocompromised hosts (5). JCPyV has been known to also cause nephropathy; however, to a much lesser extent than BKPyV (6, 7). Although there are multiple reports of JCPyV nephropathy (JCPyV-N) in KTR, there is only one previous case in the literature describing it in simultaneous heart–kidney (SHK) transplantation (8). Herein, we present a second case of JCPyV-N in a SHK transplant recipient, but the first to report JCPyV DNA-specific *in situ* hybridization (ISH) staining in kidney allograft tissue. The patient we described provided written informed consent to this report.

## Case description

**Figure 1:**
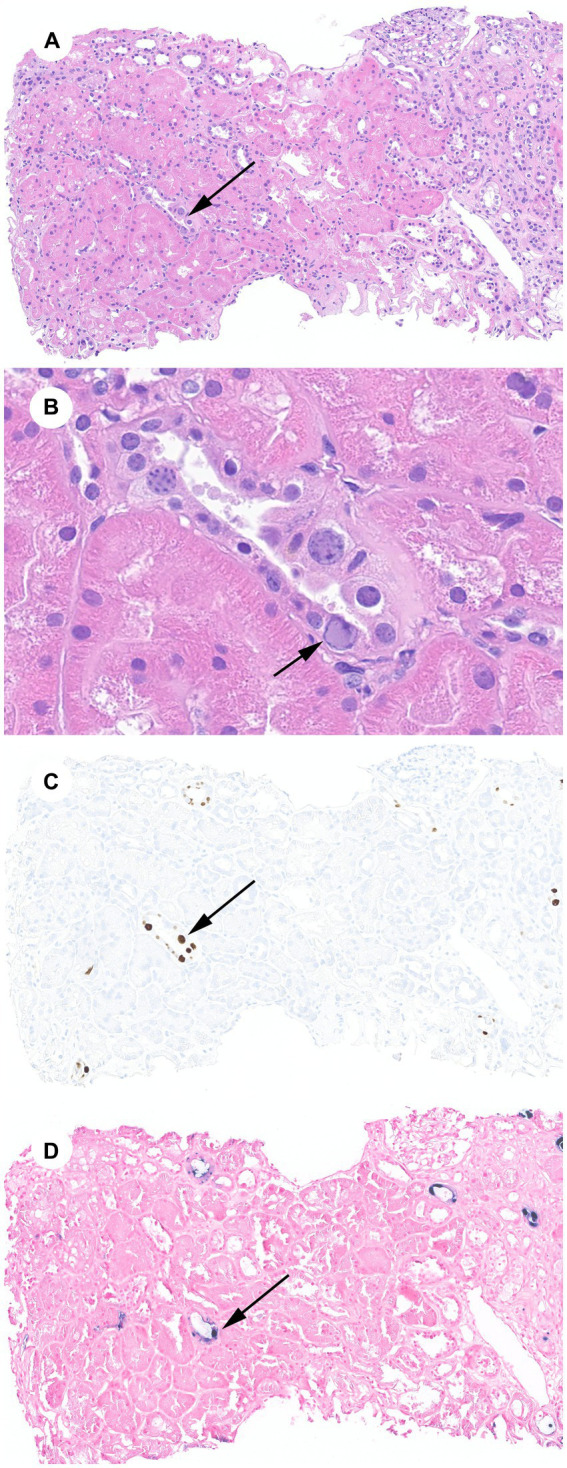
JCPyV-N: **(A)** Renal cortex with a minimal infiltrate, minimal interstitial fibrosis and tubular atrophy, and tubular epithelial cells with viral cytopathic effect (arrow) (H&E, 100X). **(B)** JCPyV cytopathic effect including glassy nuclear inclusions with chromatin margination (arrow) (H&E, 600X). **(C)** SV40 immunohistochemical stain of cytoplasmic inclusions (100X). **(D)** JCPyV ISH stain of cytoplasmic inclusions (100X).

Our patient is a 58-year-old African American man with a history of SHK transplant for a previously failed cardiac allograft and end-stage kidney disease attributed to calcineurin inhibitor toxicity based on clinical grounds. Twenty-four years before his dual-organ transplant, the patient had received an orthotopic heart transplant for non-ischemic cardiomyopathy, which subsequently failed due to graft coronary artery disease. His allograft was a Kidney Disease Profile Index of 45% deceased donor kidney with a cold ischemia time of 22 h and 30 min. The calculated panel reactive antibody was 65% and no donor-specific antibodies were present. The patient received anti-thymocyte globulin induction and mycophenolate/tacrolimus/prednisone maintenance immunosuppression per institutional protocol. This was subsequently changed to a sirolimus/lower dose tacrolimus/prednisone regimen 18 months post-transplant for concerns of calcineurin inhibitor toxicity. The patient’s infectious complications include late-onset CMV viremia 23 years after his first transplant, which was treated successfully; COVID-19 pneumonia, which required a 16 days hospitalization and was managed with supportive measures, convalescent plasma, and tocilizumab, a humanized monoclonal antibody that inhibits interleukin-6 receptor. The patient had a 2 years kidney allograft protocol biopsy that was unremarkable with no evidence of rejection, inflammation, or chronic changes, and his baseline serum creatinine (SCr) had been around 2 mg/dL. He presented 5 years and 11 months after his SHK transplant with a subacute rise in his SCr from his baseline to 2.6 mg/dL. This triggered a for-cause kidney allograft biopsy, which showed polyomavirus nephropathy with abundant SV40 immunostaining, minimal scar tissue inflammation (ti1, i0), and mild interstitial fibrosis and tubular atrophy (ci1, ct1). BKPyV DNA was undetected in both blood and urine. JCPyV DNA PCR in blood was positive at 6,052 IU/mL. Due to positive SV40 staining in the setting of JCPyV viremia and the absence of BKPyV viremia, JCPyV DNA-specific ISH staining in paraffin-embedded kidney tissue was performed and showed positive staining in the nuclei of multiple tubular epithelial cells, supporting the diagnosis of JCPyV-N (Figure 1).

**Figure 2:**
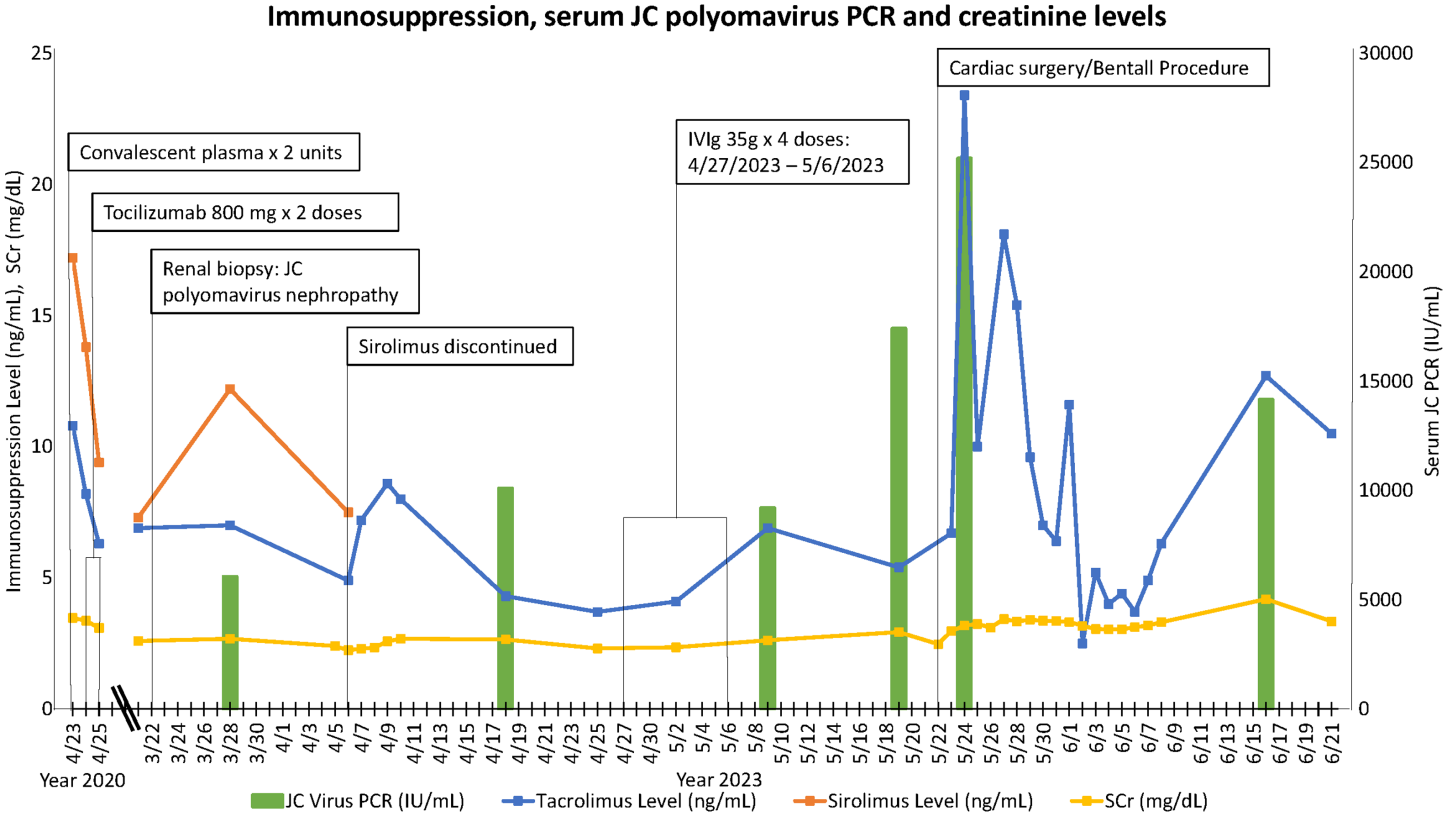
Timeline of clinical events, immunosuppression, JCPyV titer, and SCr trends.

The patient had been maintained on a tacrolimus (target trough 6–8 ng/mL)/sirolimus (target trough 3–8 ng/mL)/prednisone 5 mg daily regimen at the time of presentation. In response to his JCPyV infection, his target tacrolimus trough was reduced to 4–6 ng/mL while maintaining the sirolimus trough goal at 3–8 ng/mL. The plan was to monitor JCPyV PCR serially and titrate immunosuppression further as needed based on viral load and kidney function. One week later, the patient was admitted for an incidental finding of severe aortic insufficiency in the setting of a type-A complex dissecting aortic aneurysm involving the aortic root from the level of the sinuses of Valsalva to the mid-ascending aorta. The cardiovascular surgery team determined that the patient would require a third sternotomy for aortic root replacement with coronary artery reimplantation or bypass grafting. In anticipation of the cardiac surgery, the patient was taken off sirolimus and initiated on mycophenolate sodium 360 mg p.o. twice daily. Intravenous immunoglobulins (IVIg) 2 g/kg was administered in four divided doses for his JCPyV infection. We continued to monitor serial JCPyV PCR every 2 weeks. He underwent cardiac surgery after which he sustained acute on chronic kidney allograft insufficiency with SCr and estimated glomerular filtration rate of 3.3 mg/dL and 20 mL/min/1.73m^2^, respectively, at the time of finalizing this report. The timeline of events, immunosuppression, kidney function, and JCPyV titer trends are illustrated in Figure 2.

## Discussion

Polyomavirus infection of the genitourinary system is asymptomatic except for hemorrhagic cystitis cases that are often encountered in hematopoietic cell transplantation patients (9). Owing to this, most kidney transplant programs screen for BKPyV-N by routinely checking BKPyV PCR in the blood and/or urine every 1 to 3 months during the first 2 years post-transplantation (10). Although screening for BKPyV viremia is a common practice, screening for JCPyV is uncommon. This could be attributed to its incidence of less than 3% of all polyomavirus-associated nephropathy cases, as well as the lack of specific antiviral therapies (2). The prevalence of JCPyV seropositivity is 90%, yet the incidence of reactivation of latent infection is low. In one study, the JCPyV viremia rate was 7.6 and 6.7% in kidney and orthotopic heart transplant recipients, respectively, with about two-thirds of the cases being subclinical (11, 12). In this report, we present a case of JCPyV-N in a SHK transplant recipient. To the best of our knowledge, this is the second case report in the literature of an SHK transplant recipient and the first where JCPyV-specific ISH staining was obtained to complement SV40 staining (8).

The immunomodulatory monoclonal antibodies rituximab, natalizumab, and efalizumab, approved for the treatment of hematologic malignancies and autoimmune disorders, have been associated with PML (13). Interestingly, our patient received convalescent plasma and tocilizumab for a COVID-19 episode earlier during the pandemic. Although this exposure was 3 years before JCPyV-N diagnosis, it is plausible that COVID-19 infection itself, as well as its treatment, could have modulated his immune system and predisposed him to JCPyV-N. Moreover, maintenance immunosuppression was a more intense triple-drug regimen which is a well-known risk factor for polyomavirus infection (3). In the previous case report describing an SHK transplant recipient, the patient presented with progressive kidney dysfunction and was diagnosed with JCPyV-N 7 years post-transplant (8). Moreover, in a case series of seven KTR, JCPyV-N had an insidious presentation, and four of the subjects were diagnosed more than 6 years post-transplant (14). This is comparable to our patient, who presented 6 years post-transplant and contrasts with BKPyV-N, which is usually diagnosed within 2 years from transplantation (1–3, 15–17). The late presentation of JCPyV-N relative to BKPyV-N could be due to infectious reasons inherent to the virus itself or the delayed diagnosis given the absence of BKPyV viremia and/or viruria and thus the lack of suspicion of polyomavirus infection. Due to its delayed presentation, it may not be practical to screen for JCPyV viremia. This is because patients who are farther out from transplant surgery are usually not following closely with their transplant centers, and lab work is performed less frequently.

The negative predictive value of BKPyV viremia and, to a lesser extent, viruria in diagnosing BKPyV-N approaches 100% (8, 18). Therefore, the presence of SV40 staining and suggestive cytopathic light microscopy findings in the absence of BKPyV viremia and/or viruria should raise suspicion of JCPyV-N. JCPyV PCR in plasma should be obtained, and specific ISH staining of kidney tissue should be considered. It is possible for tissue JCPyV ISH to cross-react with BKPyV since the two viruses have 72% nucleotide homology, but it may still be beneficial to pursue if available (19). Histological changes such as tubular epithelial cell cytopathic changes, tubulointerstitial inflammation, and positive immunohistochemical staining for SV40 are common to both polyomaviruses and have no utility in distinguishing between them, albeit most published JCPyV-N cases show minimal inflammation (15). In our patient, a JCPyV-specific ISH stain was requested, which helped confirm the diagnosis of JCPyV-N (Figure 1).

Our patient was diagnosed with JCPyV-N while being evaluated for an aortopathy with type-A dissection. This required a Bentall procedure for aortic root repair, valve replacement, and coronary artery bypass grafting. Aortic tissue pathology revealed lymphoplasmacytic inflammation, the phenotype commonly seen in polyomavirus nephropathies. We therefore performed SV40 immunohistochemical staining, which was negative. This is in keeping with the lack of any existing reports of latent JCPyV infection reactivation in the form of large vessel vasculitis.

The only proven effective intervention for polyomavirus nephropathy remains to be immunosuppression reduction (1). Multiple other therapies have been proposed including cidofovir, leflunomide, quinolones, and IVIg (3, 20, 21). The inconsistent response to these various strategies has been confounded by concomitant reduction in immunosuppression. IVIg is perhaps the most commonly used second-line treatment based on some data of favorable outcomes and anecdotal evidence (21, 22). Switching patients from tacrolimus to a cyclosporine or sirolimus-based regimen has also been shown in some reports to be effective in polyomavirus nephropathy (3, 23–25). It is not certain, however, whether this is secondary to a direct antiviral activity or reduced net immunosuppression. Viral-specific cellular therapies are currently under investigation, but their use remains very limited (26). Consistent with previous reports of JCPyV-N in kidney transplant-alone recipients, we extrapolated from the BKPyV-N literature and treated our patient with immunosuppression reduction and IVIg (2). Until the time this report was finalized, we had neither seen a resolution of JCPyV viremia nor a significant improvement in kidney function (Figure 2). This could be accounted for by the inability to reduce immunosuppression further given the heart transplant status and by cardiac surgery, a major risk factor for acute kidney injury (27).

Although rare, JCPyV-N should be considered in the differential diagnosis of kidney failure in immunocompromised kidney and dual-organ transplant recipients. This is particularly important in patients with light microscopy polyomavirus nephropathy features in the absence of BKPyV viremia and viruria. Sending a plasma sample for a quantitative JCPyV PCR could be diagnostic. Confirmatory SV40 staining as well as viral-specific ISH staining of kidney tissue should be considered. If confirmed, immunosuppression reduction should be initiated promptly and followed by IVIg in refractory cases.

## Data availability statement

The original contributions presented in the study are included in the article/supplementary material, further inquiries can be directed to the corresponding author.

## Ethics statement

Written informed consent was obtained from the individual(s) for the publication of any potentially identifiable images or data included in this article.

## Author contributions

BA: Conceptualization, Supervision, Writing – original draft, Writing – review & editing. MS: Conceptualization, Writing – review & editing. MH: Conceptualization, Visualization, Writing – review & editing. GM: Conceptualization, Writing – review & editing. PB: Conceptualization, Visualization, Writing – review & editing. JR: Conceptualization, Visualization, Writing – review & editing.

## Figures and Tables

**Table 1:** 

KTR	kidney transplant recipient(s)
BKPyV	BK polyomavirus
JCPyV	JC polyomavirus
PML	progressive multifocal leukoencephalopathy
SHK	simultaneous heart-kidney
DNA	deoxyribonucleic acid
ISH	*in situ* hybridization
CMV	cytomegalovirus
COVID-19	coronavirus disease
SCr	serum creatinine
SV40	simian virus 40
ti	total interstitial inflammation score
i	interstitial inflammation score
ci	interstitial fibrosis score
ct	tubular atrophy score
PCR	polymerase chain reaction
IU	international unit
IVIg	intravenous immunoglobulin
H&E	hematoxylin and eosin staining
